# Genome wide CRISPR/Cas9 screen identifies the coagulation factor IX (F9) as a regulator of senescence

**DOI:** 10.1038/s41419-022-04569-3

**Published:** 2022-02-19

**Authors:** Paula Carpintero-Fernández, Michela Borghesan, Olga Eleftheriadou, Belen Pan-Castillo, Juan Antonio Fafián-Labora, Tom P. Mitchell, Alejandro Yuste, Muge Ogrunc, Thomas D. Nightingale, Maria Mayan, Ana O’Loghlen

**Affiliations:** 1 Epigenetics & Cellular Senescence Group; Blizard Institute; Barts and The London School of Medicine and Dentistry; Queen Mary University of London; 4 Newark Street, London, E1 2AT United Kingdom; 2 StarkAge Therapeutics, Campus de l’Institut Pasteur de Lille, 1 rue du Professeur Calmette, 59800 Lille, France; 3 Centre for Microvascular Research. The William Harvey Research Institute. Charterhouse Square Barts and the London School of Medicine and Dentistry Queen Mary University of London, EC1M 6BQ, London, UK; 4 CellCOM Research Group. Instituto de Investigación Biomédica de A Coruña (INIBIC). CH-Universitario A Coruña (XXIAC). Universidade da Coruña. Servizo Galego de Saúde (SERGAS). Xubias de Arriba, 84 15006 A Coruña, Spain; 5 SynLab Hauts-de-France, Campus de l’Institut Pasteur de Lille, 1 rue du Professeur Calmette, 59800 Lille, France; 6 Present Address: CellCOM Research Group. Instituto de Investigación Biomédica de A Coruña (INIBIC). CH-Universitario A Coruña (XXIAC), Universidade da Coruña. Servizo Galego de Saúde (SERGAS) Xubias de Arriba, 84 15006A Coruña, Spain; 7 Present Address: Grupo de investigación en Terapia Celular y Medicina Regenerativa, Departamento de Fisioterapia, Medicina y Ciencias Biomédicas, Facultad de Ciencias de la Salud, Universidade da Coruña, INIBIC-Complejo Hospitalario Universitario A Coruña (CHUAC), Agrupación estratégica CICA-INIBIC, As Xubías, 15006 A Coruña, Spain

**Keywords:** Senescence, Tumour biomarkers

## Abstract

During this last decade, the development of prosenescence therapies has become an attractive strategy as cellular senescence acts as a barrier against tumour progression. In this context, CDK4/6 inhibitors induce senescence and reduce tumour growth in breast cancer patients. However, even though cancer cells are arrested after CDK4/6 inhibitor treatment, genes regulating senescence in this context are still unknown limiting their antitumour activity. Here, using a functional genome-wide CRISPR/Cas9 genetic screen we found several genes that participate in the proliferation arrest induced by CDK4/6 inhibitors. We find that downregulation of the coagulation factor IX (*F9*) using sgRNA and shRNA prevents the cell cycle arrest and senescent-like phenotype induced in MCF7 breast tumour cells upon Palbociclib treatment. These results were confirmed using another breast cancer cell line, T47D, and with an alternative CDK4/6 inhibitor, Abemaciclib, and further tested in a panel of 22 cancer cells. While *F9* knockout prevents the induction of senescence, treatment with a recombinant F9 protein was sufficient to induce a cell cycle arrest and senescence-like state in MCF7 tumour cells. Besides, endogenous F9 is upregulated in different human primary cells cultures undergoing senescence. Importantly, bioinformatics analysis of cancer datasets suggest a role for F9 in human tumours. Altogether, these data collectively propose key genes involved in CDK4/6 inhibitor response that will be useful to design new therapeutic strategies in personalised medicine in order to increase their efficiency, stratify patients and avoid drug resistance.

## Introduction

A key characteristic of cancer cells is the deregulation of cyclin-dependent kinases (CDKs) leading to uncontrolled cell proliferation. Consequently, CDK4/6 inhibitors (Abemaciclib, Palbociclib and Ribociclib) induce senescence in vitro and in vivo [1–7]. Cellular senescence is defined as a state in which cells lose their proliferative capacity. They differ from dividing cells in terms of gene expression, chromatin structure and metabolism. Senescent cells also comprise a complex inflammatory response known as senescence-associated secretory phenotype (SASP) [8–10]. The SASP is characterised by the secretion of cytokines, enzymes, chemokines and extracellular vesicles that cause inflammation and is pivotal for the clearance of senescent cells by the immune system [11, 12]. However, the role the senescent plays during cancer is still under debate, with some studies showing its beneficial effects as a tumour suppressor mechanism, but others demonstrating it promotes tumorigenesis [8, 9, 13, 14].

CDK4/6 inhibitors induce senescence by inhibiting the phosphorylation of retinoblastoma (RB1), stabilising the RB-E2F inhibitory complex and preventing the activity of E2F transcription factors that regulate cell cycle progression [15–17]. Although initially thought to provide promising clinical outcomes due to their specificity and sensitivity for CDK4/6, resistance or intrinsic lack of response to these inhibitors has limited their clinical success [18, 19]. Thus, the molecular mechanisms by which CDK4/6 inhibitors induce senescence in cancer cells and the genes involved in conferring drug resistance or lack of response to these inhibitors are unknown, preventing patient stratification prior to Palbociclib treatment [18].

In this study, using a human genome-wide CRISPR/Cas9 library we identified genes whose loss-of-function prevent the proliferative arrest induced by Palbociclib in MCF7 breast cancer cells. Validation of the CRISPR/Cas9 screen using additional sgRNA and shRNA confirmed that among the identified genes, the coagulation factor IX (*F9*) participates in the cell cycle arrest induced by Palbociclib. These results were confirmed using Abemaciclib, where we saw that downregulation of *F9* also prevented the induction of senescence. Meanwhile, treatment with recombinant F9 induced a senescence-like proliferative arrest in MCF7 cells but not in cancer cells which did not upregulate F9 upon Palbo treatment. Our results demonstrate that F9 is endogenously upregulated upon activation of senescence by different triggers in human primary fibroblasts and endothelial cells. Finally, we screened a panel of 22 cancer cell lines for their response to different CDK4/6 inhibitors and we show that *F9* loss-of-function confers a partial resistance to the proliferative arrest induced by CDK4/6 inhibitors in other tumour types. Analyses of published datasets suggest a role for F9 in carcinogenesis in different tumours in humans. Importantly, our results open new therapeutic opportunities with the potential to stratify patients for CDK4/6 inhibitors response prior to treatment.

## Results

### A CRISPR/Cas9 genome-wide screen identifies genes overcoming the proliferative arrest induced by Palbo

**Fig. 1:**
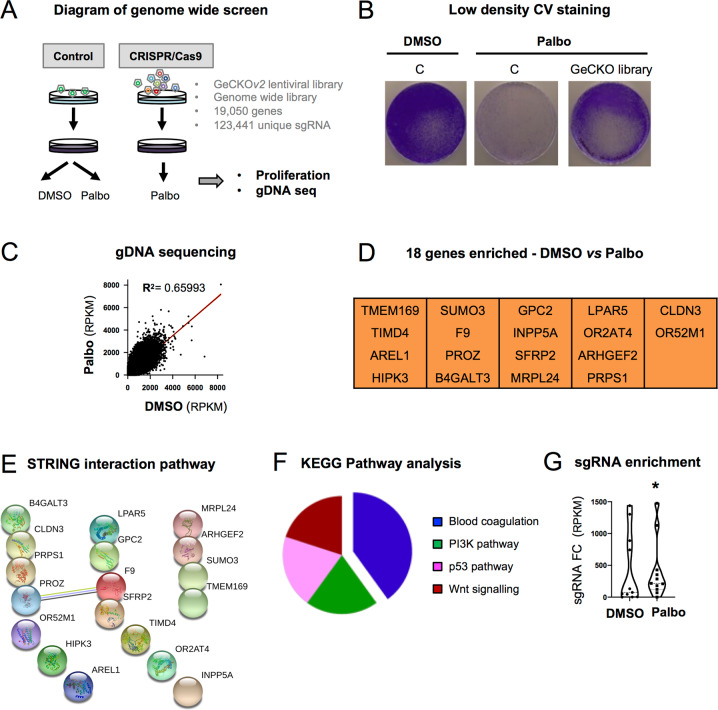
CRISPR/Cas9 screening identifies candidate genes whose loss are implicated in Palbociclib cell cycle arrest. **A** Schematic representation of the proof-of-concept genome-wide screen performed in MCF7 cells using the GeCKO*v2* pooled sgRNA library. Cells were infected with the library (CRISPR/Cas9) or the control (control), selected with puromycin and treated with 200 nM of Palbociclib (Palbo) for 14 days. **B** MCF7 cells expressing either the control (C) or the GeCKO library after 14 days of 200 nM Palbo treatment were stained with crystal violet. A representative experiment of 2 independent experiments is shown. **C** Genomic DNA (gDNA) sequencing data showing the enrichment of sgRNA after two weeks of 200 nM Palbo treatment. Data show a representative experiment from 2 independent experiments. Statistically significant (*p* < 0.05) transformed RPKM is shown. **D** sgRNA targeting 18 different genes were found to be statistically significant following the selection criteria of: (i) >2 FC (fold change) differential expression between DMSO and day 14 Palbo treatment and, (ii) 3 or more sgRNA conferring a proliferative advantage. **E** STRING protein interaction and **F** Kyoto Encyclopedia of Genes and Genomes (KEGG) analysis for the 18 genes whose sgRNA were enriched after 14 days Palbo treatment in panel D. **G** Violin plot showing all individual sgRNA within the GECKO library related to the coagulation pathway (5 sgRNAs for *PROZ* and 6 sgRNA for *F9*) enriched after two weeks Palbo treatment (FC, fold change RPKM). Median for values is shown for all sgRNA from 2 independent experiments. One sample t and Wilcoxon test was performed. Related to Fig. S1.

To identify genes whose loss-of-function overcome the proliferative arrest induced by Palbociclib (PD-0332991 or Palbo) we performed a CRISPR/Cas9 screen using the human genome-wide library GeCKO*v2* [20, 21]. This library contains ~123,441 unique sgRNA targeting 19,050 genes in the human genome with a coverage of 5–6 sgRNA per gene (Fig. 1A). To confirm that Palbo induces senescence we treated MCF7 ER^+^ breast cancer cells with increasing concentrations (0.1, 0.2, 0.5 and 1μM) for 7 and 14 days and analysed several biomarkers of senescence [11]. We confirmed that Palbo induced a stable cell cycle arrest by quantifying the number of cells staining positive for BrdU and by quantifying cell number (Fig. S1A). Palbo treatment induced an increased in β-Galactosidase activity (SA-β-Gal), a common characteristic of senescence (Fig. S1B). Neither of the doses used induced apoptosis quantified by measuring the number of cells staining positive for AnnexinV (Fig. S1C). We determined additional markers of senescence by treating MCF7 with 200 nM Palbo during 14 days and confirmed an increase in SA-β-Gal activity and in the number of cells staining positive for p21^CIP^ by immunofluorescence (IF) (Fig. S1D-F). Next, we infected MCF7 cells with a single-vector lentiviral construct (comprising sgRNA and Cas9 in a single vector), lentiCRISPRv2, (Fig. S1G) or containing the GeCKO pooled library and treated them with DMSO or 200 nM Palbo for 14 days (Fig. 1A). MCF7 cells were plated at low density where an advantage in proliferation could be observed upon the expression of the GeCKO library (Fig. 1B). Enrichment of sgRNAs after two weeks Palbo treatment compared to day 0 was determined by genomic DNA extraction and deep sequencing as previously described [20, 21] (Fig. 1C). Among all the sgRNA enriched after two weeks treatment (*p* < 0.05) we selected: (i) single sgRNA enriched ≥ 2 log_2_ fold change RPKM between day 0 and 14 and, (ii) those sgRNA where we found ≥ 3 individual sgRNA per gene preventing the proliferative arrest. A list of 18 potential genes whose loss-of-function prevent the cell cycle arrest induced by Palbo (Fig. 1D) were subjected to KEGG (Kyoto Encyclopedia of Genes and Genomes) pathway and STRING protein interaction analysis and two genes associated with the blood coagulation pathway were highlighted: the coagulation factor IX (*F9*) and Protein Z Vitamin K Dependent Plasma Glycoprotein (*PROZ*) (Fig. 1D-F). Enrichment of individual sgRNA within the GeCKO library belonging to the coagulation pathway (5 sgRNAs for *PROZ* and 6 sgRNA for *F9*) with ≥ 2 log_2_ fold change RPKM after two weeks Palbo treatment show a statistical difference (Fig. 1G). Altogether, these data propose 18 candidate genes whose loss-of-function prevent the proliferation arrest induced by Palbo with an overrepresentation of two genes involved in the blood coagulation pathway.

### *F9* and *PROZ* loss-of-function prevent the proliferation arrest induced by Palbo

**Fig. 2:**
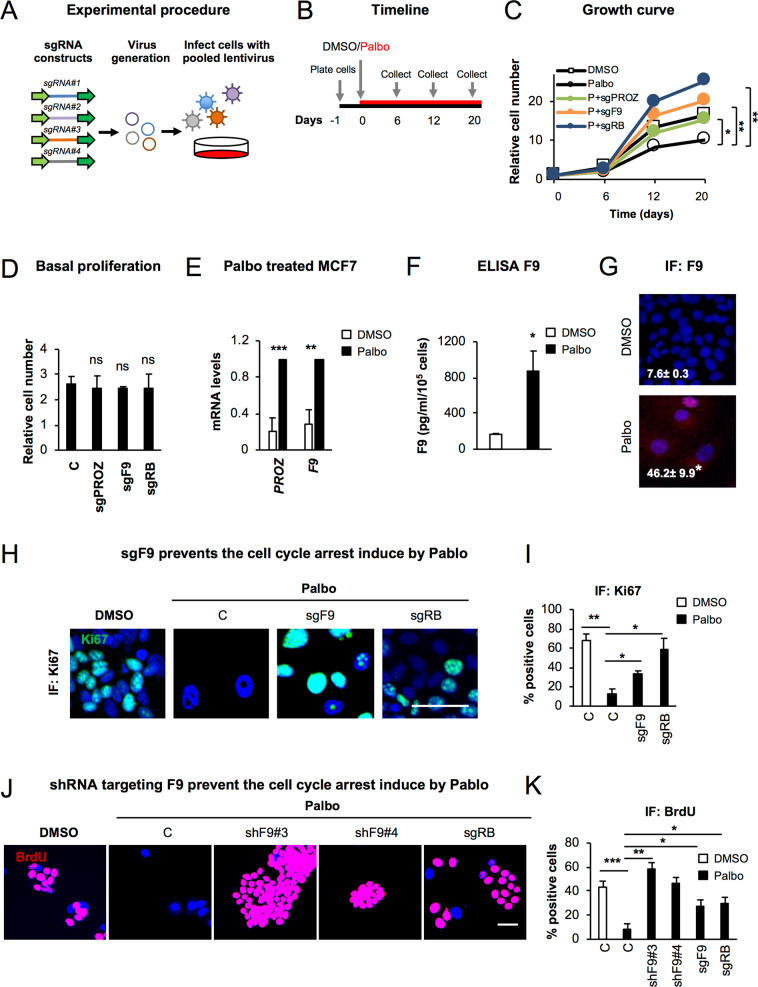
CRISPR/Cas9 screen validation identifies *F9* as a regulator of the proliferation arrest induced by Palbo. **A** Schematic representation of the viral production for the CRISPR/Cas9 screen validation. 4 sgRNA targeting the same gene (e.g. PROZ or F9) were pooled and used to infect MCF7 cells. **B** Overview of the experimental set-up followed to validate the identified sgRNA. Briefly, after plating, MCF7 infected cells were treated with 200 nM Palbo and samples were collected to determine cell number at days 6, 12 and 20 after Palbo treatment. **C** MFC7 cells expressing the indicated sgRNAS were treated with 200 nM Palbo for 20 days and collected at different time points (0, 6, 12, 20 days) to asses proliferation. Proliferation curves show that MCF7 expressing sgF9 (orange line) and sgPROZ (green line) prevented a stable cell cycle arrest compared to Palbo (P) treated cells (black line - circles). sgRB MCF7 cells treated with Palbo (blue line) were used as a positive control. The data represent the mean of 4 independent experiments for C, Palbo and sgPROZ and 3 for sgF9 and sgRB. Student’s t-test analysis at day 20 was performed compared to the Palbo treated sample. **D** Basal proliferation rate was determined by quantifying nuclei count after sgRNA infection at 20 days of cell culture. Data show the mean ± SEM of 3 independent experiments for C, sgF9 and 2 for sgPROZ and sgRB. Two-tailed student’s t-test compared to the C sample was performed. **E** MCF7 treated with Palbo for 20 days induce an upregulation of *F9* and *PROZ* mRNA levels as shown by qPCR analysis. Data represent the mean ± SEM of 5 independent experiments. Two-tailed t-test analysis was performed. **F** ELISA for F9 protein levels secreted by MCF7 cells upon DMSO or Palbo treatment for 20 days. Data represent the mean ± SEM of 4 independent experiments. Two-tailed t-test was performed. **G** Representative images and quantification for F9 staining in MCF7 cells treated with 500 nM Palbo for 7 days. Data represent the mean ± SEM of 3 independent experiments. Two-tailed t-test was performed. **H**, **I** (**H**) Representative images and **I** quantification showing that sgF9 prevents the proliferation arrest induced by Palbo by displaying an increase in the percentage of cells staining positive for Ki67 (green). Data show the mean ± SEM of 3 independent experiments. Two-tailed t-test analysis comparing to Palbo sample was performed. Scale bar: 50μm. **J**, **K** (**J**) Representative images and **K** quantification for BrdU staining of MCF7 cells infected with a construct expressing two individual shRNA targeting F9 (shF9#3 and shF9#4). sgF9 and sgRB are used as a positive control. Scale bar: 50μm. Data show the mean ± SEM of 3 independent experiments. Two-tailed students t-test compared to the Palbo sample was performed. See also Fig. S2.

To validate the implication of *F9* and *PROZ* we designed and cloned 4 additional sgRNA targeting *PROZ* (sgPROZ) or *F9* (sgF9) (Fig. 2A). We included an sgRNA targeting *RB1* (sgRB) as a control [22]. MCF7 cells were infected with 4 pooled sgRNAs targeting each candidate gene and treated with DMSO or 200 nM Palbo. The ability of sgF9, sgPROZ and sgRB to prevent the proliferation arrest induced by Palbo was confirmed by performing proliferation curves at different days after treatment (Fig. 2B, C). The efficacy of the sgRNAs were assessed at the mRNA (Fig. S2A) and protein level for RB (Fig. S2B). Importantly, MCF7 basal proliferation was not affected by sgF9 or sgPROZ expression alone, confirming that the bypass in proliferation was specific to sgF9 and sgPROZ upon Palbo treatment (Fig. 2D). Next, we hypothesised that *F9* and *PROZ* were upregulated during senescence, and thus, their loss-of-function was the cause of the lack of response to Palbo. Indeed, we could observe an increase in the mRNA expression levels of *F9* and *PROZ* in MCF7 cells after 20 days Palbo treatment (Fig. 2E). To determine whether F9 could be secreted as part of the SASP, we performed an ELISA assay and confirmed an increase in the amount of F9 released upon Palbo treatment (Fig. 2F). Furthermore, an increase in the protein levels of F9 could also be detected by IF (Fig. 2G).

We next wanted to corroborate that the proliferation arrest upon Palbo treatment was stable and that both sgPROZ and sgF9 were implicated in bypassing this arrest. MCF7 cells were treated with Palbo for 6 days, washed and cultured in the absence of the drug until day 20 (Fig. S2C). As shown in Fig. S2D treatment of MCF7 cells with Palbo resulted in a stable inhibition of proliferation even in the absence of the drug as shown by low-density plating and CV staining (Fig. S2D).

### *F9* loss-of-function prevents the senescence-like phenotype induced by Palbo

We decided to focus on *F9* knockout as its proliferation bypass is stronger than sgPROZ (Fig. 2C). We thus determined whether this proliferative advantage was maintained using different concentrations of Palbo. We treated MCF7 cells with 200 nM and 500 nM Palbo for 20 days and counted cell numbers at day 20 (Fig. S2E). The knockout efficiency of sgF9 and sgRB was validated at day 20 to ensure that plasmid expression was not lost (Fig. S2F). sgF9 bypass was also shown by quantifying the percentage of cells staining positive for Ki67 by IF (Fig. 2H, I) and low-density cell plating (Fig. S2G), with no changes in the apoptosis marker AnnexinV (Fig. S2H).

We next tested whether sgF9 could be inducing an increase in the migration capacity of MCF7 cells, thus promoting tumorigenesis. However, we show a decrease in the relative number of MCF7 cells migrating upon 500 nM Palbo treatment for 20 days which is not prevented by sgF9 (Fig. S2I). MDA-MB-468 cells were used as a control.

To exclude the potential implication of off-target effects derived from using CRISPR/Cas9, we infected MCF7 cells with two independent viral constructs carrying an shRNA targeting *F9* (shF9#3 and shF9#4). Both shF9 constructs recapitulated the effects observed with sgF9 measured by quantifying the number of cells staining positive for BrdU (Fig. 2J, K). *F9* downregulation was confirmed at the mRNA level by qPCR (Fig. S2J). Altogether, these data show that *F9* loss-of-function using genome editing and RNAi interference overcomes the cell cycle arrest induced by Palbo in MCF7 cells.

### F9 is endogenously upregulated during senescence

**Fig. 3:**
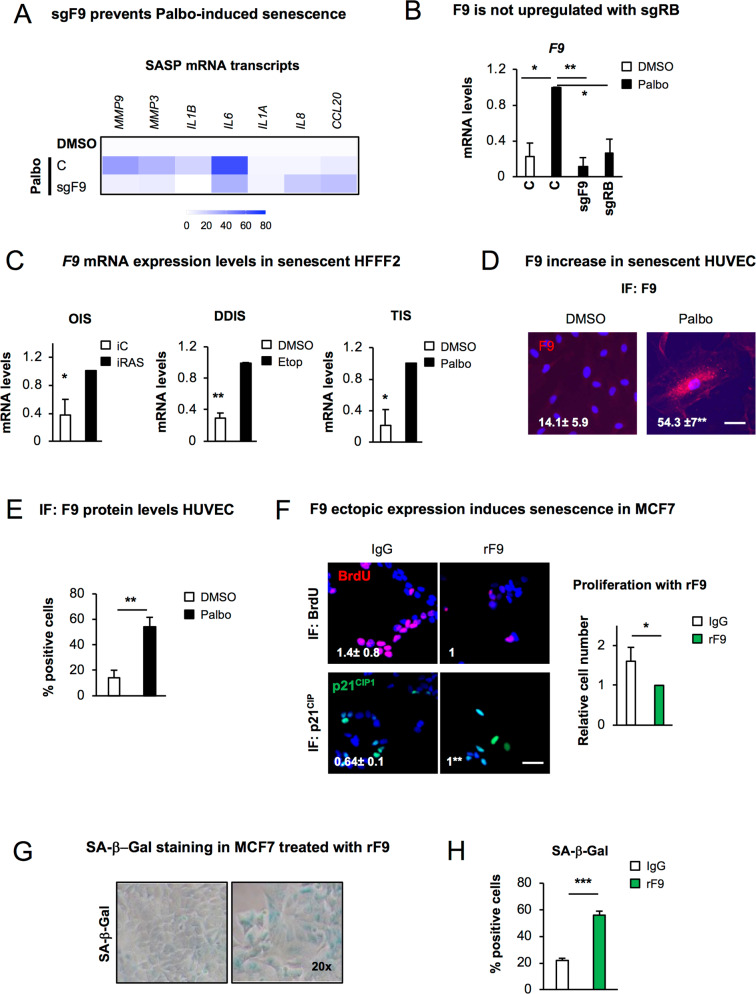
F9 induces senescence and it is endogenously upregulated during senescence. **A** Heat map of SASP mRNA levels in MCF7 cells control or expressing sgF9 after 20 days treatment with Palbo. The mean of 2–7 independent biological replicates is shown. **B** *F9* mRNA upregulation by Palbo is prevented when RB is not present (sgRB). Data show the mean ± SEM of 4 independent experiments. One-WAY ANOVA with Dunnett’s multiple comparison to Palbo sample was performed. **C** mRNA levels of endogenous *F9* mRNA levels in HFFF2 (human primary fibroblasts) upon the induction of senescence. OIS (Oncogene-induced senescence) was induced in HFFF2 expressing ER:H-RAS^G12V^ (iRAS) by adding 200 nM 4OHT for 6 days (left panel); 50μM etoposide was added for 2 days and washed out until day 7 to induce DDIS (DNA-damage senescence) (middle panel); TIS (Therapy-induced senescence) was mimicked by treating with 1μM of Palbo for 7 days (right panel). Data represent the mean ± SEM of 3 independent experiments for OIS and DDIS and 2 for TIS. Two-tailed student’s t-test analysis was performed. **D** Representative immunofluorescence images and **E** IF quantification showing the expression of endogenous F9 (red) in HUVEC (human umbilical vein endothelial cells) upon 7 days treatment with 500 nM Palbo. Scale bar: 50μm. The data represent the mean ± SEM of 4 independent experiments. Two-tailed student´s t-test was used to calculate statistical significance. **F Left panel**, Representative images for BrdU (red) and p21^CIP1^ (green) in MCF7 treated twice with 10μg/mL of recombinant F9 (rF9) for 6 days. Scale bar: 50 μm. 3 independent experiments were performed. **Right panel**, The graph shows a reduction in the number of cells at the end of the experiment. Mean ± SD of 3 independent experiments is shown. Two-tailed student´s t-test was used to determine statistical significance. **G** Representative pictures and **H** quantification of MCF7 cells staining positive for SA-β-Gal activity when treated twice with 10μg/mL rF9. Data represent mean ± SEM of 3 independent experiments. Two-tailed student´s t-test was used. See also Fig. S3.

We set to determine the importance of F9 expression during senescence; thus, we evaluated whether sgF9 prevented the induction of other features of senescence such as the SASP. sgF9 downregulates several SASP mRNA transcripts upregulated by Palbo such as *MMP9*, *MMP3*, *IL1B* and *IL6* while having no effect on *CCL20*, *IL1A* or *IL8* (Fig. 3A). Importantly, sgRB averted the endogenous upregulation of *F9* mRNA levels (Fig. 3B), suggesting that *F9* mRNA expression is dependent on RB.

Next, we took advantage of human primary fibroblasts (HFFF2) expressing an endoplasmic reticulum (ER):H-RAS^G12V^ fusion protein (iRAS) where 200 nM 4-hydroxytamoxifen (4OHT) treatment for 6 days induces senescence [6, 12]. In fact, we observe a consistent upregulation of endogenous *F9* mRNA during OIS (Fig. 3C) concomitant with an increased SA-β-gal activity (Fig. S3A). This was recapitulated in HFFF2 treated with 50μM etoposide for 2 days and collecting RNA at day 7, mimicking DNA-damage-induced senescence (DDIS). Moreover, treatment of HFFF2 fibroblasts with 1μM Palbo for 7 days, mirroring therapy-induced senescence (TIS), also triggered the endogenous upregulation of *F9* mRNA (Fig. 3C). Confirmation of the induction of senescence was further demonstrated by showing a reduction in cell proliferation measured by quantifying cell number and an increase in the number of SA-β-gal positive cells (Fig. S3B, C) [6, 12, 23]. Activation of senescence and endogenous upregulation of F9 was also observed in human umbilical vein endothelial cells (HUVEC). HUVEC cells treated with 500 nM Palbo for 7 days showed an increase in the percentage of cells staining positive for F9 by IF (Fig. 3D, E) concomitant with reduced proliferation, an increase in p21^CIP1^ and an increase in the levels of secreted F9 (Fig. S3D-F).

Finally, ectopic administration of 10μg/mL recombinant F9 protein (rF9) twice during 6 days induced a senescence-like phenotype in MCF7 cells shown by a reduction in cell numbers, a decrease in BrdU incorporation, an increase in p21^CIP^ positive cells associated with increased SA-β-Gal activity (Fig. 3F-H). Interestingly, treatment of the melanoma cell line SKMEL28, which does not upregulate *F9* upon Palbo treatment, (Fig. S3G) failed to induce a significant increase in p21^CIP^ upon rF9 treatment in spite of showing a proliferation arrest as presented in Fig. S3H.

Altogether, these data highlight the implication of F9 in inducing senescence using a variety of triggers in different human primary cell cultures and the implication of the induction of senescence upon treatment with rF9.

### *F9* regulates senescence in MCF7 cells treated with Abema and in T47D

**Fig. 4:**
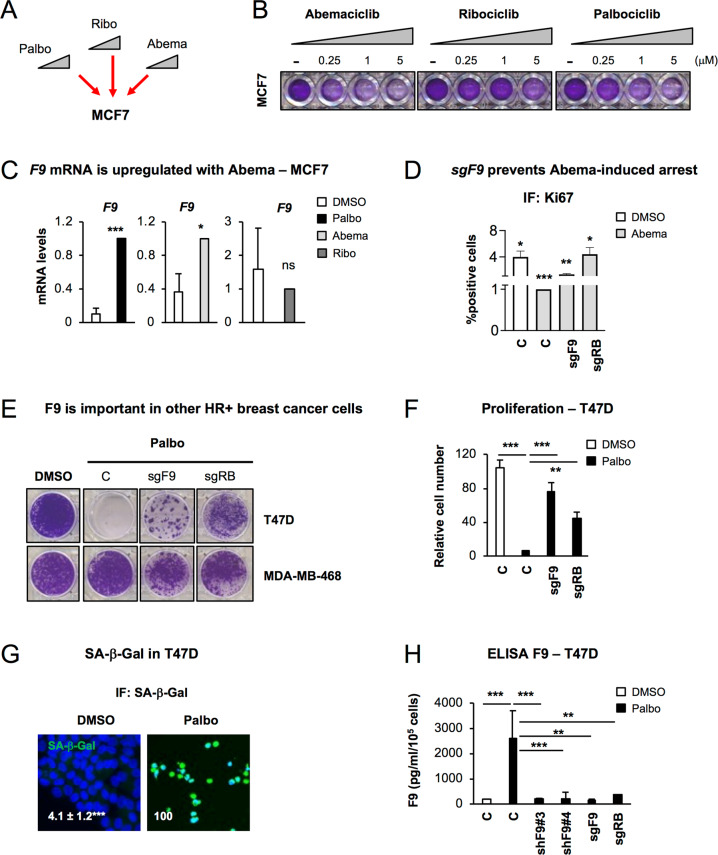
CDK4/6 inhibitors induce a cell cycle arrest in different tumour types and is dependent on F9 in T47D cells. **A** Schematic representation of the treatment of MCF7 cells with 3 different CDK4/6 inhibitors: Palbociclib (Palbo), Ribociclib (Ribo) and Abemaciclib (Abema). **B** Colony formation assay stained with crystal violet for MCF7 treated with increasing concentrations of different CDK4/6 inhibitors for 10 days. A representative experiment is shown. **C** qPCR data show an upregulation of endogenous *F9* mRNA levels upon treatment with 500 nM Palbo, 500 nM Abema or 500 nM Ribo. Data show the mean ± SEM of 3 independent experiments. Two-tailed *t-*test analysis is shown for statistical significance. **D** Ki67 proliferation assay in MCF7 cells expressing sgF9 or sgRB treated with 1μM Abema for 20 days. Representative experiment of 3 independent experiments is shown. Quantifications shows mean ± SEM. Wilcoxon test analysis performed. **E** Crystal violet staining shows the effect on proliferation in T47D (ER^+^ breast cancer cells) and MDA-MB-468 (triple negative breast cancer cell line) expressing sgF9 or sgRB and treated with Palbo. Representative staining of 4 independent experiments is shown. **F** Relative cell count for T47D cells expressing either sgF9 or sgRB. Data show the mean ± SEM of 7 independent experiments. One Way ANOVA with Dunnett’s multiple comparisons to Palbo C sample was performed. **G** Representative pictures and quantification for number of cells presenting SA-β-Gal activity in T47D treated with 1μM Palbo for 20 days. Two-tailed student’s t-test was performed. Scale bar: 50 μM. **H** ELISA for human F9 released to the conditioned media in T47D cell treated with 1μM Palbo for 20 days expressing or not F9. sgRB is used as positive control. Data show mean ± SEM of 3 independent experiments Two-tailed student’s t-test was performed. Related to Fig. S4.

Next, we wanted to determine whether the proliferation bypass by sgF9 was due to the specific inhibition of CDK4/6 and not due to off-target effects. For this, we used increasing concentrations (0.25, 1 and 5μM) of Abemaciclib (Abema) or Ribociclib (Ribo), including Palbo as a positive control (Fig. 4A, B). We could observe a dose-dependent cell cycle arrest upon treatment with other CDK4/6 inhibitors (Abema in particular) concomitant with a decrease in RB protein levels (Fig. 4B, Fig. S4A) which was maintained after 14 days of continuous treatment (Fig. S4B, C**)**. We next confirmed the induction of a stable cell cycle arrest characteristic of senescence by treating MCF7 cells for 6 days with Abema, Palbo or Ribo, withdrawing the inhibitors and leaving for 14 further days after the drug removal (Fig. S4D). Our data confirm that both Palbo and Abema induce a stable cell cycle arrest even after drug withdrawal which was not maintained when the cells were treated with Ribo (Fig. S4D). In line with our previous results, *F9* mRNA levels increased when MCF7 cells were treated with Palbo and Abema but not with Ribo (Fig. 4C). We treated sgF9 MCF7 cells with 1μM Abema for 20 days and show that it partially prevented the cell cycle arrest by Ki67 (Fig. 4D) and by crystal violet staining (Fig. S4E). Furthermore, we determined the implication of sgF9 using another ER^+^ (T47D) and the triple-negative (MDA-MB-468) breast cancer cell lines. sgF9 prevented the cell cycle arrest induced by Palbo in T47D cells as shown by colony formation assay (Fig. 4E) and by counting cell numbers (Fig. 4F). The knockout efficiency for *RB* and *F9* was determined at the protein level for RB (Fig. S4F) and RNA levels for *RB* and *F9* (Fig. S4G**)**. However, MDA-MB-468 cells did not respond to Palbo treatment as previously reported [15] (Fig. 4E) in spite of *F9* and *RB* being downregulated (Fig. S4H) by their respective sgRNAs. The induction of senescence by Palbo in T47D was further confirmed by measuring SA-β-Gal activity (Fig. 4G) and the release of F9 by ELISA (Fig. 4H). The implication of sgF9 was further validated using two independent shF9#3 and shF9#4 by colony formation assay (Fig. S4I), relative cell count (Fig. S4J) and F9 secretion (Fig. 4H). Confirmation of the knockdown efficiency was confirmed by qPCR (Fig. S4K). Altogether, these data show that F9 loss prevents the induction of senescence by treatment with Palbo and Abema in T47D cells.

### Other cancer cell lines respond to CDK4/6 inhibitors and induce *F9* upregulation

**Fig. 5:**
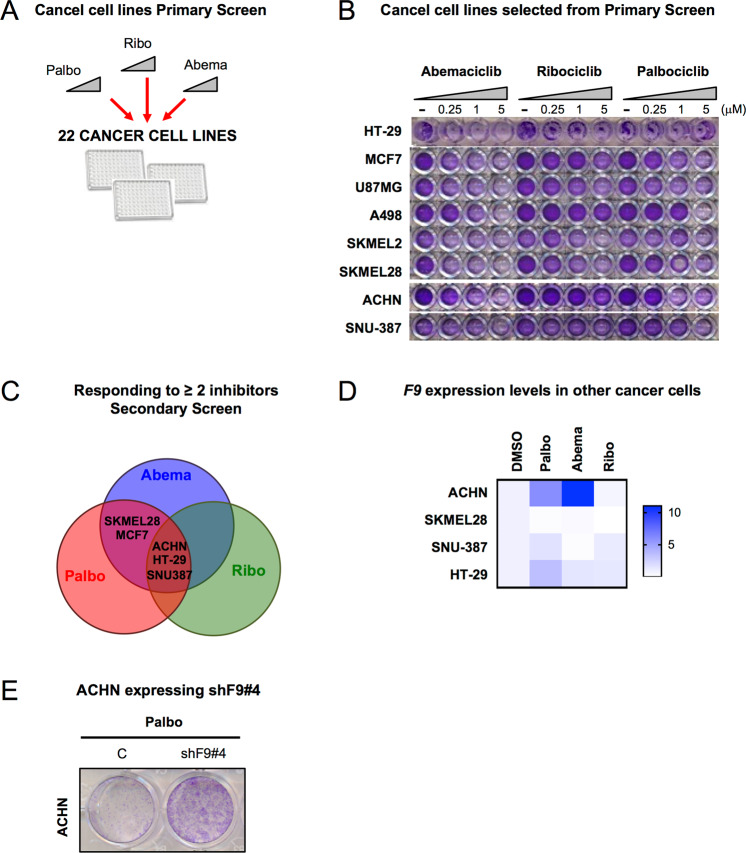
CDK4/6 inhibitor response in a panel of different cancer cell lines. **A** A panel of 22 cancer cell lines of different origins were treated with increasing concentrations of Palbo, Ribo and Abema. **B** Crystal violet staining showing the 8 cancer cell lines that responded in a statistically significant (*p* < 0.05) and dose-dependent manner to more than 2 inhibitors. Representative experiment is shown. **C** Venn diagram shows that SKMEL28 (melanoma), MCF7 (breast cancer), ACHN (renal adenocarcinoma), HT-29 (colon) and SNU-387 (liver) cancer cell lines responded to two or more CDK4/6/ inhibitors (*p* < 0.05) in a Secondary Screen and were selected for further validation. **D** Heat map showing *F9* mRNA expression in the indicated cancer cells after treatment with different CDK4/6 inhibitors. The map represents the mean of 3–5 independent replicates. **E** ACHN control or expressing shF9#4 stained with crystal violet after 20 days treatment with Palbo show a partial proliferation bypass. See also Fig. S5.

To further determine if there is a wider implication for *F9* loss-of-function in other types of cancers, we tested a panel of 22 cancer cell lines from different origins and molecular characteristics with increasing concentrations of Palbo, Abema and Ribo (Fig. 5A, Fig. S5A). 8 cancer cell lines (including MCF7) responded to ≥ 2 CDK4/6 inhibitors in a dose-dependent and statistically significant manner (*p* < 0.05) (Fig. 5B). Further validation of these 8 cells lines in a secondary screen re-evaluating the proliferation arrest induced by all three CDK4/6 inhibitors confirmed the response of 5 cell lines (*p* < 0.05) to ≥ 2 CDK4/6 inhibitors (MCF7, SKMEL28, ACHN, HT-29, SNU-387) (Fig. 5C, Fig. S5B, C). Next, we determined which of these 5 cell lines (excluding MCF7) induced an upregulation of endogenous *F9* mRNA levels upon the treatment with all three inhibitors by qPCR. Of all the cancer cell lines analysed, the renal adenocarcinoma cell line (ACHN) was the only cell line to upregulate *F9* with Palbo and Abema (Fig. 5D), while the human colorectal adenocarcinoma cell line (HT29) and the hepatocellular carcinoma cell (SNU-387) increased *F9* mRNA expression only with Palbo (Fig. 5D). *F9* was not upregulated by Ribo in any of the cell lines analysed (Fig. 5D). A partial proliferation bypass by crystal violet staining in ACHN upon shF9#4 expression was observed (Fig. 5E). This bypass could implicate that *F9* is only partially important in Palbo induced senescence in ACHN cells and that other mechanisms might be implicated. Altogether, our data highlight a partial relevance for *F9* mRNA expression in other cancer cell lines.

### *F9* is highly expressed in the tumour stroma

**Fig. 6:**
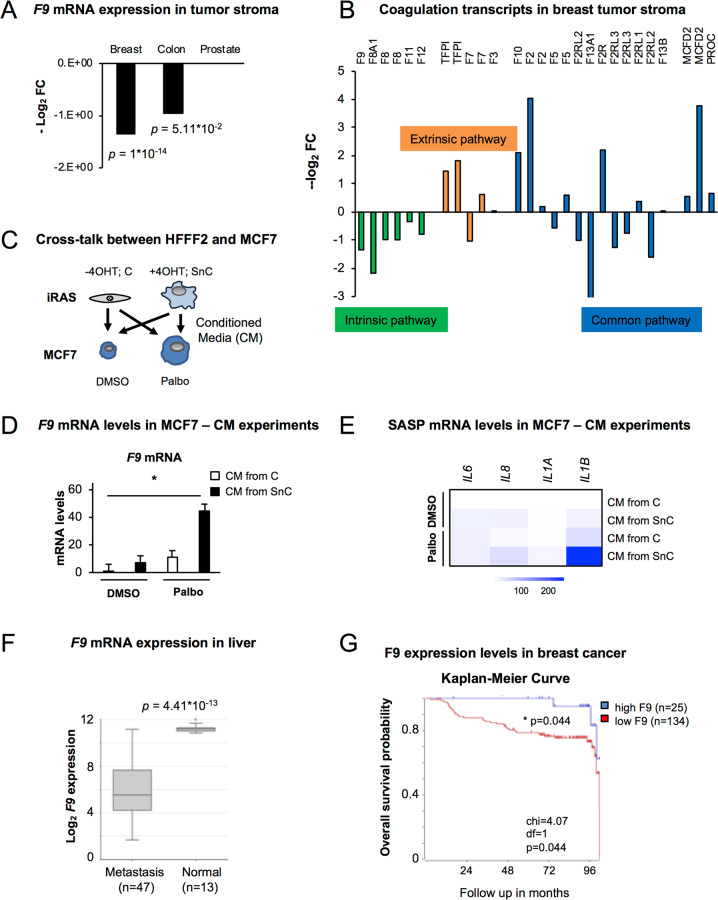
*F9* expression is important in different tumours. **A** *F9* mRNA levels in tumour stroma *vs* healthy stroma in breast, colon and prostate cancers. Data are represented as -log_2_ fold change (FC) from [27]. Breast (*n* = 12 samples from normal *versus* *n* = 111 from tumour); colon (*n* = 4 samples from normal *versus* *n* = 13 from tumour); prostate (*n* = 10 samples from normal versus *n* = 8 from tumour). T-test student analyses is performed. **B** mRNA expression levels of different mRNA transcripts from genes implicated in the intrinsic coagulation pathway (green bars), extrinsic coagulation pathway (orange bars) and transcripts common to both pathways (blue bars) in breast cancer. Comparison of tumour stroma (*n* = 111) *vs* healthy stroma (*n* = 12) [27]. **C** Schematic representation of panels **D** and **E**. MCF7 cells pre-treated with DMSO or 500 nM Palbo were incubated for 72 h with the conditioned media (CM) of control (-4OHT) (C) or senescent (+4OHT) (SnC) iRAS HFFF2 primary fibroblasts. Senescence was induced with 200 nM 4OHT for 3 days, washed, incubated with fresh media and collected after 3 additional days. **D** *F9* mRNA levels and **E** heatmap for other SASP transcripts in MCF7 pre-treated with DMSO or 500 nM Palbo and incubated with the CM from iRAS (-/+ 200 nM 4OHT; C or SnC) for 72 h. Data show the mean ± SEM of 4 independent replicates for *F9* and the mean of 4 independent experiments for the SASP. Two-way ANOVA with Dunnett’s multiple comparisons analyses was performed. **F** F9 mRNA expression levels in liver from normal (*n* = 13 samples) and metastatic (*n* = 47) liver. One-Way ANOVA analysis was performed to determine statistical significance. Dataset was calculated using R2. **G** Kaplan-Meier survival curve for high (blue) (*n* = 25) or low (red) (*n* = 134) F9 expression levels and overall survival prognostic in breast cancer. Chi-square = 4.07; *p* = 0.04 [30]. Dataset calculated with R2.

Next, we sought to explore public datasets of gene expression in the tumour stroma of several human cancers: GSE34312 (prostate) [24], GSE9014 (breast) [25], and GSE35602 (colon) [26]. We found that *F9* is upregulated in the tumour stroma in comparison with healthy stroma in breast and colon, but not in prostate cancer (Fig. 6A). When analysing other transcripts implicated in the intrinsic coagulation pathway where F9 is involved, most are also upregulated in breast cancer, while transcripts within the extrinsic pathway are mainly downregulated (Fig. 6B) [27]. As the tumour microenvironment is composed of cancer cells and stroma, we next sought to identify whether a cross-talk existed between iRAS fibroblasts and MCF7 treated with DMSO or Palbo (Fig. 6C). Senescence was induced in iRAS cells with 200 nM 4OHT for 3 days, washed and incubated with fresh media after which the conditioned media (CM) was collected (Fig. 6C). MCF7 pre-treated with or without 500 nM Palbo for 7 days plus 3 days in 0.5% serum were incubated with the CM from iRAS for 72 h and we determined changes in *F9* mRNA levels (Fig. 6D). Interestingly, we found a sharp increase in *F9* mRNA levels when senescent MCF7 cells (pretreated with Palbo) were incubated with the CM of senescent iRAS (+4OHT). This increase also correlated with other SASP mRNA transcripts (Fig. 6E) confirming previous studies highlighting that the SASP reinforces the cell cycle arrest and the induction of senescence [28]. The SASP of MCF7 cells (which express F9 upon the induction of senescence) also reinforced the senescence of Palbo-treated MCF7 and T47D (Fig. S6A, B). In contrast, when two cancer cell lines that did not upregulate F9, SKMEL28 (Fig. 5D) and MDA-MB-468 (Fig. S6C) were treated with the conditioned media of Palbo-treated MCF7 or iRAS cells, no differences could be observed in the levels of *F9* mRNA (Fig. S6D) in spite of *F9* mRNA levels being upregulated upon 4OHT treatment in iRAS cells (Fig. S6E). The same results could be observed upon treatment of SKMEL28 with the CM from MCF7 underoing Palbo treatment or not (Fig. S6F). Altogether, these results highlighting the relevance of F9 as a regulator of senescence induced by Palbo.

### *F9* loss-of-function is associated with metastasis and worse survival prognostics in cancer

Our data suggest that increased levels of *F9* upon the induction of senescence maintain certain cancer cells in a stable cell cycle arrest, while *F9* loss-of-function promotes a proliferative advantage. In accordance with our data, low levels of *F9* are associated with liver metastasis [29] (Fig. 6F). Importantly, high levels of F9 expression are a sign of good prognostic for survival in breast cancer [30] (Fig. 6G). Interestingly, low expression levels of F9 in different subtypes of breast cancer (basal, luminal A and luminal B) are also associated with poor prognosis and lower disease-free survival probability (Fig. S6G, I). Altogether, these data show the potential of using F9 levels as a biomarker for patient stratification not only to predict response to CDK4/6 inhibitors but also as a prognostic marker to determine overall survival in breast cancer.

## Discussion

A better understanding of the mechanisms regulating senescence induced by CDK4/6 inhibitors is needed in order to increase therapy efficacy and patient stratification in cancer. In this study, by performing a whole genome-wide CRISPR/Cas9 screen, we have unveiled a role for the coagulation factor IX (*F9*) as an important regulator of senescence induced by CDK4/6 inhibitors in ER^+^ breast cancer cell lines and other cancer cells, thus identifying genes whose loss can predicted a lack of response to Palbo.

Previous publications have shown that inflammaging, hypercoagulability and cellular senescence share common pathways [31]. In fact, a correlation between thrombosis and cancer [32–34] is well known, although the link with cellular senescence is less characterised. Interestingly, Wiley et al. demonstrated that human fibroblasts undergoing senescence secrete a subset of haemostasis-related factors as part of the SASP [35]. It is however curious to note that they did not find factors implicated in the coagulation pathway suggesting that maybe different triggers or cell types could favour either general haemostasis or the coagulation pathway specifically. Importantly, the combination of MEK and CDK4/6 inhibitors in pancreatic adenocarcinoma induce senescence including the release of SASP factors enriched in pro-angiogenic proteins promoting tumour vascularisation and enhancing drug delivery and efficacy [7]. In line with these findings, senescent human primary fibroblasts also release pro-angiogenic factors such as VEGF [36]. Altogether, these findings, in conjunction with ours, show that *angiogenesis* and the coagulation pathway play an important role in senescence. In fact, the coagulation pathway has been shown to be upregulated in senescence [37] and ageing [38, 39]. Furthermore, F9 has been associated with frailty [40] and is a genetic risk factor estimated to contribute to thrombosis incidence in the elderly [38, 41].

The expression of the oncogene *MET* in the liver in vivo causes hepatocarcinogenesis, blood hypercoagulation and internal bleeding [32]. In fact, the use of antiplatelet therapy in liver cancer prevents cross-talk between platelets and immune cells [42]. Curiously, chemotherapy treatment increases the risk of thrombosis [43] but it also induces senescence promoting cancer relapse [13]. Importantly, F9 expression can induce regulatory CD4^+^ T cells in mice suggesting a role for F9 in immunosurveillance [44]. It would therefore be interesting to further explore the implications of the coagulation pathway and F9 in the context of chemotherapy and senescence immunosurveillance.

In summary, here we provide evidence for the involvement of two different genes within the coagulation pathway, *F9* and *PROZ*, in regulating senescence in different contexts. Our results show that *F9* is partially responsible for CDK4/6 induced senescence. Importantly, we unveiled a correlation between the levels of F9 and cancer progression studying different cancer-related datasets. Altogether, we believe *F9* could be considered as a potential biomarker to predict CDK4/6 inhibitor response when used as first-line treatment in cancer.

## Experimental procedures

### Cell culture

All cancer cell lines used in this study were obtained from American Type Culture Collection (ATCC) and were not authenticated upon arrival. Mycoplasma testing was performed. Human foreskin fibroblasts (HFFF2) were obtained from Culture Collections (Public Health England, UK). All cell lines were grown in high glucose Dulbecco’s Modified Eagle Medium (DMEM) (Gibco) except for HCT-116, SK-OV-3, Capan2 and HT29 that were grown in McCoys 5a medium, SNU-387, NCI-H23, OVCAR-3 that were grown in RPMI and A549, PC-3 that were grown in F12K medium. All media was supplemented with 10% foetal bovine serum (FBS) (Thermo Fisher) and 1% of antibiotic-anti-mycotic (Thermo Fisher). Human umbilical vein endothelial cells (HUVEC) from pooled donors were purchased from Promocell (Heidelberg, Germany). The cells were grown in M199 (Life Technologies, Grand Island, NY) with 20% foetal bovine serum (Labtech, Heathfield, UK), 10U/ml heparin (Sigma, St. Louis, MO), and 30 μg/ml endothelial cell growth supplement (Sigma). Experiments were performed using HUVECs between passage 3 and 5.

### Senescence induction

MCF7 cells were treated with different concentrations (100 nM, 200 nM, 500 nM or 1000 nM) of Palbociclib (PD0332991) (APExBIO), Abemaciclib (LY2835219) (Selleckchem) or Ribociclib (LEE011) (Selleckchem) and the other cancer cell lines with the indicated concentration of inhibitor. Media was changed every 2 days. Human primary fibroblasts (HFFF2) were treated with 50μM of Etoposide (Sigma-Aldrich) for 2 days and cells collected at day 7 or treated with 1μM Palbociclib for 7 days. HFFF2 expressing pLNC-ER:RAS vector, were induced to senescence by adding 200 nM 4-hydroxytamoxifen (4OHT) (Sigma-Aldrich) for 6 days. All treatments were done using DMEM supplemented with 10% FBS and 1% of antibiotic-anti-mycotic. HUVEC cells were treated with 500 nM Palbo for 7 days to induce senescence.

### F9 recombinant experiments

Recombinant experiments were carried out by using F9 recombinant protein (R&D systems). MCF7 or SKMEL28 cancer cell lines were treated with 10 μg/ml rF9 in complete medium for 72 hours with 2 treatments in total (6 days treatment). At the end of the experiment, cells were fixed with 4% paraformaldehyde (PFA) and used for immunofluorescence studies or stained for SA-β-Gal activity.

### Retroviral and lentiviral infections

The generation of stable retroviral and lentiviral expression was carried out following previous studies [6, 12, 23, 45]. Briefly, retroviral particles were generated by transfecting pLNC-ER:H-RAS^G12V^ plasmid and retroviral helper plasmids (vsvg and gag-pol) with Polyethylenimine (PEI) in HEK293T packaging cells for 48 h. Recombinant lentiviral particles were generated using the second-generation packaging vectors psPAX2 and pMD2.G using PEI in HEK293T. The supernatant containing retrovirus or lentivirus was then filtered with 0.45 µm filters (Starlab) and applied to HFFF2 cells in the presence of 4 µg/ml polybrene (hexadimethrine bromide; Sigma-Aldrich) following 3 rounds of infection. Cells were subsequently selected with the appropriate antibiotic resistance either 0.5 μg/ml puromycin or 300 μg/ml neomycin (Invitrogen). For lentiviral infections with the sgRNA a pool for the 4 sgRNA (5μg DNA per single sgRNA) targeting a single gene was generated by transfecting equal amounts of DNA and the packaging vectors psPAX2 and pMD2.G. Infection was performed as described earlier for lentivirus.

### Genome-wide CRISPR/Cas9 library amplification and sequencing

The Human CRISPR knockout Pooled Library (GeCKO*v2*) was purchased from Addgene (#1000000048) and amplified using *E. coli* competent cells. The library contains 123,411 unique sgRNA sequences targeting 19,050 genes within the human genome. In order to maintain library representation and sufficient sgRNA coverage the library was amplified in large culture dishes (24 cm^2^) obtaining an approximate transformation efficiency of 4 × 10^8^ equivalent to ~10.000 colonies per sgRNA construct. After amplifying the library as described [20, 46], viral particles were produced in HEK293T cells and MCF7 cells were infected at a multiplicity of infection (MOI) of 0.2–0.5 following the lentiviral protocol previously described [20, 46]. Cells were selected with puromycin (1 μg/ml) for 72 h after the GeCKO library infection. After selection, cells were plated at low density and treated with 200 nM Palbociclib for 14 days to either determine proliferation or sent DNA for genomic DNA sequencing. Crystal violet staining was used to assess cell proliferation and determine the library bypass efficacy. After MCF7 cell infection and selection with the GeCKO library, genomic DNA was extracted at days 0 and day 14 after infection using the QIAmp Blood and Cell Culture DNA midi kit (Qiagen). The PCR was performed by QMUL Genome Centre. Analysis of genomic DNA at say 0 showed an sgRNA coverage higher than > 88%. The accession number for the sequencing data reported in this paper is GEO: GSE192525.

### CRISPR sgRNA generation

The online guide design tool (http://crispr.mit.edu) was used to identify sgRNA sequences. The highest scoring guides were selected. Primers for the sgRNA sequences were ordered and the complementary sequences annealed at 37 C for 30 min, followed by incubating the annealed primers at 95 C for 5 min and then ramped down to 25 C at 5 C degrees per min. The annealed synthetic sgRNA oligonucleotides were cloned into pLentiCRISPR*v2* vector (Addgene #52961) at BsmBI restriction sites. The sgRNA sequences used in this study are:

sgF9#1GCAGCGCGTGAACATGATCATGG

sgF9#2CACTGAGTAGATATCCTAAAAGG

sgF9#3ATGATCATGGCAGAATCACCAGG

sgF9#4CTAAAAGGCAGATGGTGATGAGG

sgLPAR5#1CCCAGAGGGCTAGCGCGTTGAGG

sgLPAR5#2CCAGAGGGCTAGCGCGTTGAGGG

sgLPAR5#3GGAAGATGGCGCCCGTCGTCTGG

sgLPAR5#4GCGTAGTAGGAGAGACGAACGGG

sgPROZ#1TGAGGGCTCCACACGATGGAGGG

sgPROZ#2GGTCCTCGCCCTCCATCGTGTGG

sgPROZ#3CTGAGGGCTCCACACGATGGAGG

sgPROZ#4GCTCCACACGATGGAGGGCGAGG

sgMOGAT#1CCGCAATGTAGTTCCGAGAGGGG

sgMOGAT#2GCCCGCAATGTAGTTCCGAGAGG

sgMOGAT#3GTTCCGCAGTAACAGCGTGAAGG

sgMOGAT#4GCTGTTACTGCGGAACCGAAAGG

sgRB#3GGTGGCGGCCGTTTTTCGGGGGG

sgRB#4CGGCGGTGGCGGCCGTTTTTCGG

To identify positive clones the primer hU6 CRISPR5’-GAGGGCCTATTTCCCATGATT-3’ was used in combination with the reverse primer for each specific clone and isolated clones were SANGER sequenced.

### Cell proliferation experiments

For cell proliferation studies, 100 cells were plated in each well of a 96-well plate and treated with CDK inhibitors (Palbociclib, Abemaciclib, Ribociclib) for 0, 6, 15, 20 days. The medium was replaced every other day either with drug or drug-free medium. For replating experiments, cells were treated with Palbociclib for 6 days, counted and replated at low density in a 96 well plate until day 20. Drug-withdrawal was performed by treating the cells 6 days in the presence of Palbociclib and removing the drug from day 6 to day 20. Cells were then fixed, stained with 0.5% crystal violet, solubilized with 30% acetic acid solution and absorbance was measured at 570 nm.

For colony formation assay, 5000 cells were plated in each well of a six-well plate, treated with CDK inhibitors 20–30 days (once control reached confluence). Cells were then washed with PBS, stained with crystal violet and scanned to obtain the pictures.

### CDK4/6 inhibitors dose-response studies

Dose-response studies were carried out using the panel of cancer cell lines listed in the Cell Culture section. Cells were plated in a 96 well plate at 500–1000 cells/well (based on seeding density calculations) and treated with increasing concentrations (0.25–5 μM) of Palbociclib, Abemaciclib and Ribociclib. Medium containing the drugs or DMSO was replaced every other day during 10 days. The plates were then stained with 0.5% crystal violet solution and scanned. Crystal violet quantification was performed by solubilizing crystal violet staining with 30% acetic acid and measuring the absorbance at 570 nm.

### β-galactosidase staining

Cells were washed with PBS and fixed with 0.05% (w/v) glutaraldehyde (in PBS) for 15 min at room temperature. Cells were washed a second time with PBS and incubated with 5-bromo-4-chloro-3-indolyl-beta-D-galacto-pyranoside (X-gal) solution for 1 h at 37 °C. Cells were imaged after 12–24 h using a light microscope (Nikon) at 20X magnification and single representative images of each well were taken. Fluorescent β-Galactosidase was performed according to the manufacturer’s instructions using the following commercial kit (Sigma-Aldrich, #F2756). Briefly, 33 μM of the β-gal substrate C_12_FDG (Fluorescein di-β-D-galactopyranose) (F2756 Sigma-Aldrich) was added to the cells for 8 h at 37 °C, After, the cells were washed with PBS and fixed with 4% PFA.

### RNA extraction, cDNA synthesis and qPCR

Total RNA was extracted using TRIzol Reagent (ThermoFisher) according to the manufacturer’s instructions. cDNA synthesis was performed using High Capacity cDNA Reverse Transcriptase kit (ThermoFisher). qPCR reactions were performed using SYBR Green PCR Master Mix (Applied Biosystems) on a 7500 Fast System RealTime PCR cycler (Applied Biosystems). Primer sequences used in this study are:

F9 Forward5′-CAGTGTTCAGAGCCAAGCAA-3′

F9 Reverse5′-CATGGTGAACACGAAACCAG-3′

PROZ Forward5′-CACCCCTGAGAAAGACTTCG-3′

PROZ Reverse5′-GGAGCCTCTGTGTTCTCTGG-3′

RB Forward5′-AACCCAGGAAGGAATGGCT-3′

RB Reverse5′-CTGCGTTCAGGTGATTGATG-3′

IL8 Forward5′-GAGTGGACCACACTGCGCCA-3′

IL8 Reverse5′-TCCACAACCCTCTGCACCCAGT-3′

IL6 Forward5′’-CCAGGAGCCCAGCTATGAAC-3′

IL6 Reverse5′-CCCAGGGAGAAGGCAACTG-3′

CCL20 Forward5′-GGCGAATCAGAAGCAGCAAGCAAC-3′

CCL20 Reverse5′-ATTGGCCAGCTGCCGTGTGAA-3′

IL1A Forward5′-AGTGCTGCTGAAGGAGATGCCTGA-3′

IL1A Reverse5′-CCCCTGCCAAGCACACCCAGTA-3′

IL1B Forward5′-TGCACGCTCCGGGACTCACA-3′

IL1B Reverse5′-CATGGAGAACACCACTTGTTGCTCC-3′

CDKN1A Forward5′-CCTGTCACTGTCTTGTACCCT-3′

CDKN1A Reverse5′-GCGTTTGGAGTGGTAGAAATCT-3′

ACTIN Forward5′-GCCCTGAGGCACTCTTCCA-3′

ACTIN Reverse5′-CGGATGTCCACGTCACACTTC-3′

RSP14 Forward5′-CTGCGAGTGCTGTCAGAGG-3′

RSP14 Reverse5′-TCACCGCCCTACACATCAAACT-3′

### Protein lysis and western blot

Cells were lysed in ice-cold Lysis Buffer 6 (R&D systems) supplemented with 10 μL/mL of protease inhibitor cocktail. Total protein content was determined by Precision Red Reagent (Sigma) protein assay. Twenty micrograms of total protein were separated on 10% SDS-PAGE and transferred to a polyvinylidene fluoride (PVDF) membrane (Millipore Co., Bedford, MA). Protein transfer was checked by staining the membrane with Ponceau S red (Sigma-Aldrich). The membrane was then blocked using 5% bovine serum albumin (BSA) (Sigma) or 5% milk (Sigma) in PBS supplemented with 0.05% Tween-20 (Sigma) (PBST). Primary antibodies RB1 (BD; Cat# 554136) and β-Actin (Abcam, Cat# ab8226) were incubated overnight at 4 C. After four washes with PBST, the membrane was incubated with a secondary antibody for 1 h at room temperature. Protein bands were detected using SuperSignal West Pico PLUS Chemiluminescent Substrate (Thermo Fisher Scientific) using the ChemiDoc XRS + System (Bio-Rad).

### Immunofluorescence

Cells were grown in a 96-well plate and fixed with 4% paraformaldehyde for 10 min at RT. Cells were then washed twice with PBS and permeabilized by incubating with 0.4% Triton X-100 in PBS for 10 min at RT. After a PBS wash, cells were blocked 30 min at RT using 1% BSA in PBS supplemented with 0.1% Tween-20 (PBST). Primary antibodies (details found at the end of the section) were diluted in 1% BSA-PBST and incubated overnight. For BrdU staining, cells were treated with DNaseI and MgCl_2_ simultaneously with the primary antibody. Cells were then washed with PBS and incubated with their respective secondary antibody for 1 h at RT. Nuclei were stained with DAPI (Sigma-Aldrich). Images were acquired using INCell 2200 automated 991 microscope (GE) and INCell 2200 Developer software version 1.8 (GE) was used for image analysis. Antibodies used in this study are: p21^CIP^ (Abcam, Cat# ab109520), Ki67 (Abcam, Cat# ab92742), BrdU (Abcam, ab6326; 1:500) and F9 (Proteintech, Cat# 21481–1-AP).

### Conditioned media experiments

Donor cells (HFFF2 iRAS) were treated in the presence or absence of 200 nM 4-hydroxytamoxifen (4OHT) (Sigma-Aldrich) for 3 days, washed and replenished with fresh media to prevent carrying the 4OHT. Conditioned medium (CM) was collected and supplemented to 10% FBS and added to MCF7, T47D, SKMEL28 or MDA-MB-468 recipient cells for 72 h. MCF7 or MDA-MB-468 cells were pre-treated in the presence or absence of 500 nM Palbociclib prior to adding the CM.

When using MCF7 as donors, cells were treated in the presence or absence of 500 nM Palbociclib for 7 days. At day 7 Palbo was removed and DMEM 0.5% FBS was added for 72 h to collect the Conditioned medium (CM). CM was collected and supplemented to 10% FBS and added to recipient cells (MCF7, T47D, SKMEL28) for 72 h. Recipient cells were pre-treated prior to CM addition in the presence or absence of 500 nM Palbociclib.

### ELISA

Quantitative analysis to determine the presence of Human Factor 9 was performed by evaluating the concentration of F9 in the conditioned media of different cell cultures using the Abcam Human Factor IX ELISA Kit (ab108831). The amount of F9 was normalised to cell number at the end of each experiment.

### Statistics

Dataset analysis were performed using R2: Genomics Analysis and Visualization Platform (http://r2.amc.nl). STRING interactions were identified using the functional protein association networks (https://string-db.org/). Kyoto Encyclopedia of Genes and Genomes (KEGG) pathway analysis was performed using Panther pathway analysis (http://www.pantherdb.org). All statistical analyses performed are specified in the figure legend. A *p* < 0.05 is considered significant throughout the paper as follows: *p < 0.05; **p < 0.01; ***p < 0.001.

## Supplementary information

Reproducibility File

Supplemental Material
