# Supplementary material for: Genome wide CRISPR/Cas9 screen identifies the coagulation factor IX (F9) as a regulator of senescence
Source: Cell Death Dis. 2022 Feb 19;13(2):163. doi: 10.1038/s41419-022-04569-3 (PMC8858321; doi:10.1038/s41419-022-04569-3)
Supplement: Supplementary file 2 — Supplemental Material [file 41419_2022_4569_MOESM2_ESM.pdf]

Figure S1

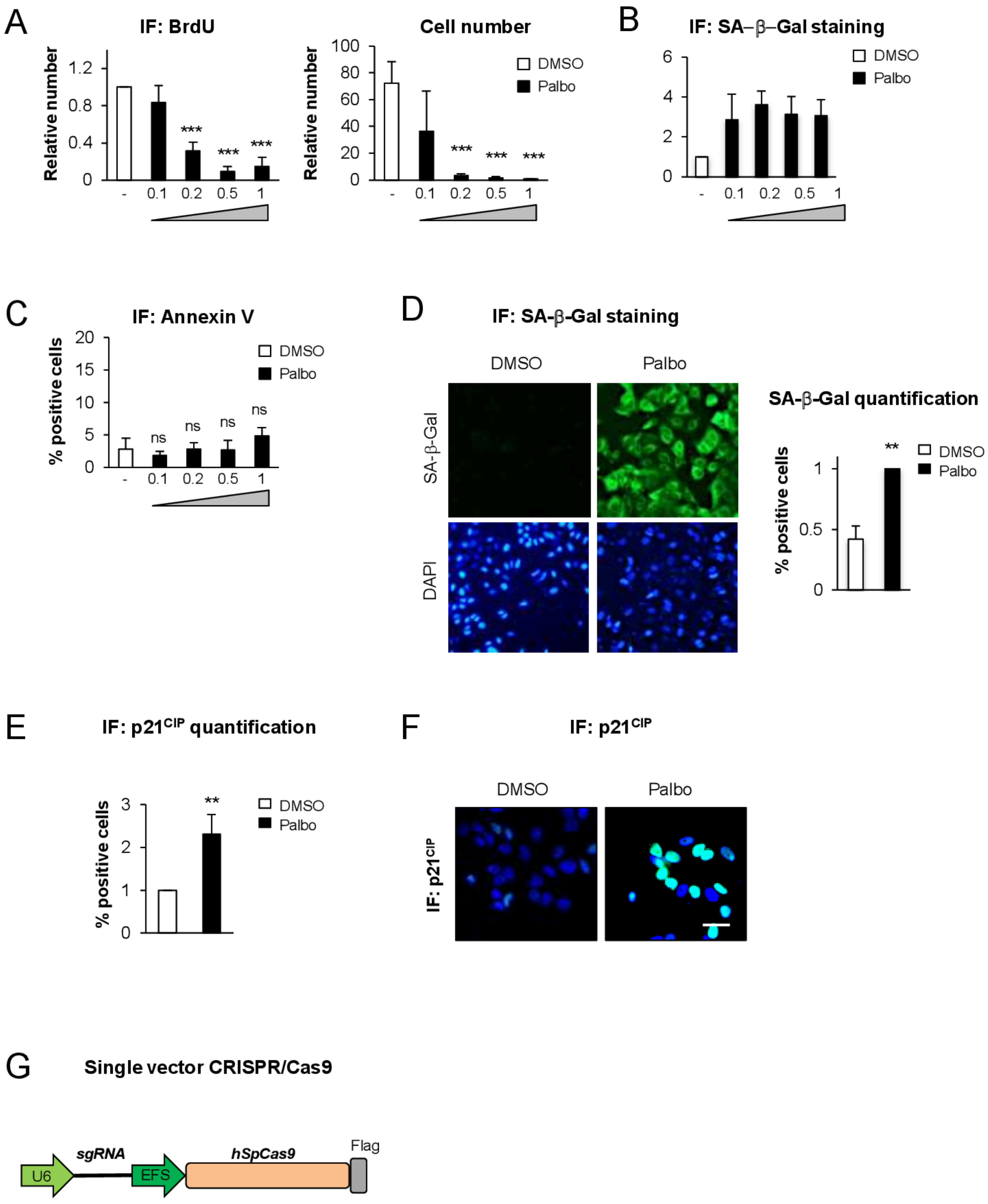
A
IF: BrdU
Relative number
1.2
0.8
0.4
0
- 0.1 0.2 0.5 1
***
Cell number
100
80
60
40
20
0
DMSO
Palbo
B
IF: SA–β–Gal staining
6
4
2
C
IF: Annexin V
% positive cells
20
15
10
5
ns
D
IF: SA-β-Gal staining
SA-β-Gal
DAPI
SA-β-Gal quantification
**
0.5
E
IF: p21CIP quantification
3
F
IF: p21CIP
G
Single vector CRISPR/Cas9
U6
sgRNA
EFS
hSpCas9
Flag

Figure S1. Palbociclib induces a senescent-like phenotype in MCF7 breast cancer cell line. (A) Quantification of BrdU incorporation and relative cell number in MFC7 treated with different concentrations (0.1, 0.2, 0.5 and 1 μM) of Palbociclib (Palbo) for 14 days. **(B)** SA-β-galactosidase (SA-β-Gal) staining in MCF7 cells treated with different concentrations of Palbo for 7 days. **(C)** The graph represents the percentage of Annexin V positive cells after 14 days Palbo treatment with different concentrations. **(D)** Representative images (left panel) and quantification (right panel) for SA-β-Gal staining in MCF7 cells treated with 200nM Palbo for 14 days. Graph shows the mean ± SEM of 4 independent experiments. Two-tailed Student´s t-test was used to calculate statistical significance. **(E)** Quantification and **(F)** representative pictures of the percentage of $p21^{CIP}$ positive cells upon 14 days 200nM Palbo treatment. Two-tailed Student´s t-test was used to calculate statistical significance. Scale bar: 50 μm. **(G)** Diagram for the lentiCRISPR*v2* one vector system used in the GeCKO library and to clone individual sgRNAs. The plasmid contains an expression cassette for human Cas9 (*hSpCas9*) and the sgRNA in the same vector. Related to **Figure 1**.

Figure S2

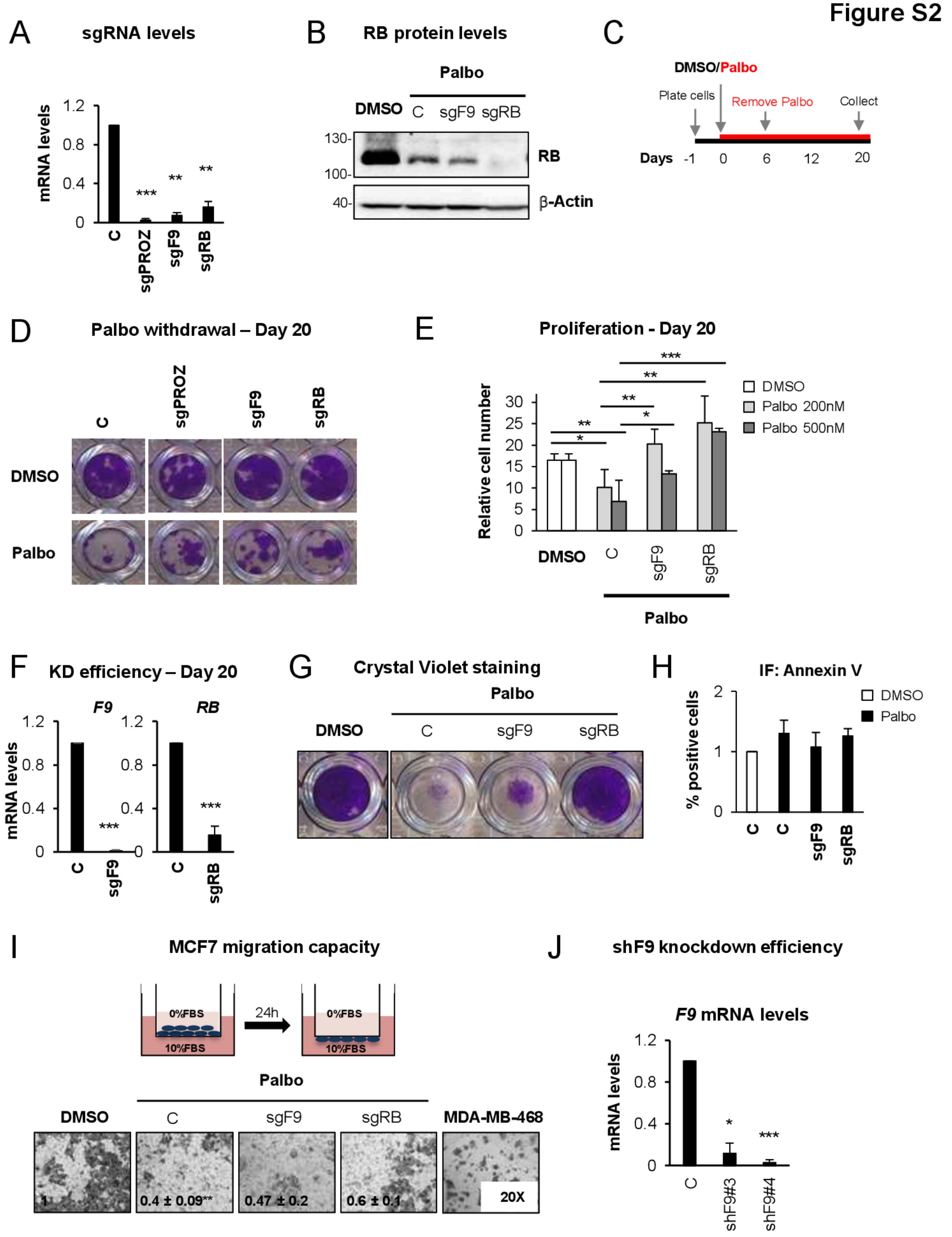
A
sgRNA levels
mRNA levels
1.2
0.8
0.4
0
C
sgPROZ
sgF9
sgRB
***
**
**
B
RB protein levels
Palbo
DMSO
C
sgF9
sgRB
130-
100-
40-
RB
β-Actin
C
DMSO/Palbo
Plate cells
Remove Palbo
Collect
Days
-1
0
6
12
20
D
Palbo withdrawal – Day 20
C
sgPROZ
sgF9
sgRB
DMSO
Palbo
E
Proliferation - Day 20
Relative cell number
30
25
20
15
10
5
0
DMSO
Palbo 200nM
Palbo 500nM
DMSO
C
sgF9
sgRB
Palbo
F
KD efficiency – Day 20
F9
RB
mRNA levels
1.2
0.8
0.4
0
C
sgF9
C
sgRB
***
***
G
Crystal Violet staining
Palbo
DMSO
C
sgF9
sgRB
H
IF: Annexin V
% positive cells
2
1
0
DMSO
Palbo
C
C
sgF9
sgRB
I
MCF7 migration capacity
0%FBS
10%FBS
24h
0%FBS
10%FBS
Palbo
DMSO
C
sgF9
sgRB
MDA-MB-468
1
0.4 ± 0.09**
0.47 ± 0.2
0.6 ± 0.1
20X
J
shF9 knockdown efficiency
F9 mRNA levels
mRNA levels
1.2
0.8
0.4
0
C
shF9#3
shF9#4
*
***

Figure S2. Validation of F9 loss-of-function efficiency and proliferative advantage in MCF7 cells. (A) Relative mRNA expression levels of *PROZ*, *F9* and *RB* in MCF7 after their respective sgRNA infection and selection. Graph shows the mean ± SEM of 2 independent experiments. Two-tailed Student´s t-test was used to calculate statistical significance compared to the Control sample. **(B)** Representative western blot showing RB knockout upon sgRB expression in MCF7 cells. β-actin was used as a loading control. Representative blot for 4 independent experiments. **(C)** Timeline and strategy followed to confirm that the senescence proliferative arrest is maintained after Palbo is removed and washed out. MCF7 cells were treated with DMSO or 500nM Palbo for 6 days, after which the drug was removed and the cells were grown and collected at day 20. **(D)** Crystal violet staining showing the proliferation rate of MCF7 cells expressing sgRNAs (sgPROZ, sgF9 and sgRB) at day 20. Palbo was removed after day 6 to confirm the induction of a stable cell cycle arrest. A representative experiment of 3 independent experiments is shown. **(E)** Relative cell number quantification in MCF7 control or expressing sgRNAs (sgF9, sgRB) using 200nM (light grey) or 500nM (dark grey) Palbo treatment for 20 days. Data represent the mean ± SEM of 3 independent experiments. **(F)** mRNA levels for *F9* and *RB* by qPCR in MCF7 cells 20 days after setting the experiment. Data represent the mean ± SD of 3 independent experiments. Two-tailed Student´s t-test was used to calculate statistical significance. **(G)** Crystal violet staining for MCF7 expressing sgF9 and treated with 500nM Palbo. A representative experiment is shown. sgRB is used as positive control. **(H)** Quantification for AnnexinV staining in MCF7 cells expressing sgF9 and sgRB upon Palbo treatment. Data represent the mean ± SEM of 3 independent experiments. **(I)** 24h MCF7 migration assays upon 500nM Palbo treatment for 20 days expressing sgF9 and sgRB. MDA-MB-468 cells were used as a positive control. Representative pictures of 3 independent experiments is shown. Data show the mean ± SEM of 3 independent experiments. One-Way ANOVA with Dunnett's multiple comparisons to Palbo was performed. **(J)** qPCR to confirm the efficacy of two independent shRNA targeting F9

(shF9#3 and shF9#4). Data represent the mean ± SEM. Two tailed students t-test analysis was performed. Related to **Figure 2**.

Figure S3

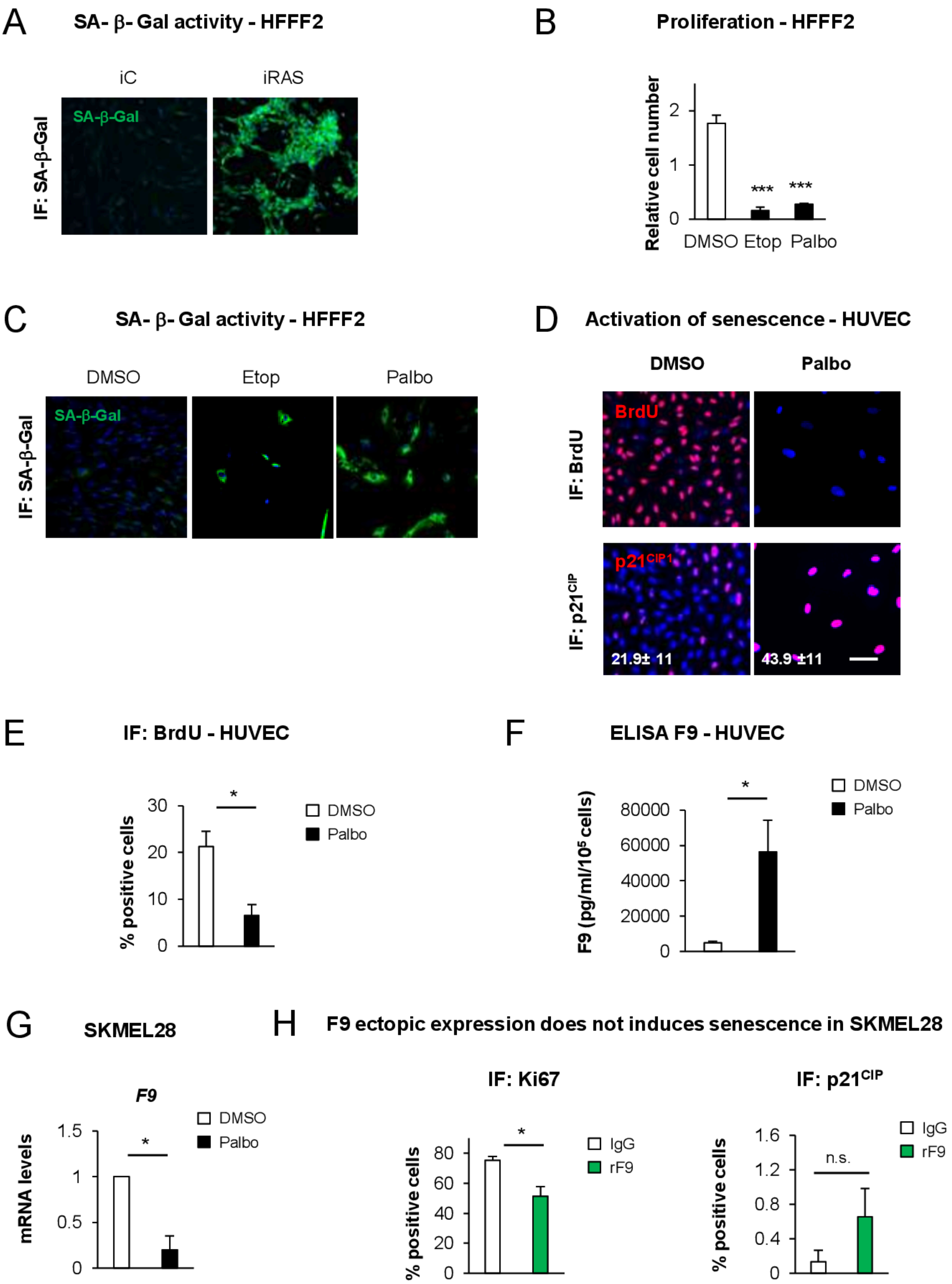
A
SA- β- Gal activity - HFFF2
iC
iRAS
IF: SA-β-Gal
SA-β-Gal
B
Proliferation - HFFF2
Relative cell number
0
1
2
***
***
DMSO
Etop
Palbo
C
SA- β- Gal activity - HFFF2
DMSO
Etop
Palbo
IF: SA-β-Gal
SA-β-Gal
D
Activation of senescence - HUVEC
DMSO
Palbo
IF: BrdU
BrdU
IF: p21CIP
p21CIP1
21.9± 11
43.9 ±11
E
IF: BrdU - HUVEC
% positive cells
0
10
20
30
*
DMSO
Palbo
F
ELISA F9 - HUVEC
F9 (pg/ml/10⁵ cells)
0
20000
40000
60000
80000
*
DMSO
Palbo
G
SKMEL28
F9
mRNA levels
0
0.5
1
1.5
*
DMSO
Palbo
H
F9 ectopic expression does not induces senescence in SKMEL28
IF: Ki67
% positive cells
0
20
40
60
80
*
IgG
rF9
IF: p21CIP
% positive cells
0
0.4
0.8
1.2
1.6
n.s.
IgG
rF9

Figure S3. Evaluation of senescence in different human primary cultures and additional cancer cell lines. (A) Representative images of SA-β-galactosidase activity (SA-β-Gal). Human primary fibroblasts (HFFF2) expressing an empty vector ER:EV (iC) or ER:H-RAS^{G12V} (iRAS) were treated with 200nM 4OHT for 6 days to induce senescence. SA-β-Gal is shown by the incorporation of the fluorescent compound C_{12}FDG (green). **(B)** HFFF2 relative cell number after 2 days treatment with 50 μM of Etoposide followed by 5 days with fresh media or 7 days with 1μM Palbociclib. Data show the mean ± SEM of 3 independent experiments. Two-tailed students t-test was performed compared to Control sample. **(C)** HFFF2 treated with 50μM of Etoposide for 2 days followed by 5 days with fresh media and 1μM Palbo for 7 days were incubated for 8h with C_{12}FDG compound. SA-β-gal activity (green) was determined by fluorescent signal and representative images are shown. **(D)** Representative IF images for p21^{CIP1} and BrdU in HUVEC (human umbilical vein endothelial cells) control or treated with 500nM Palbo for 7 days. Representative images are shown from 3 independent experiments for BrdU and 4 for p21^{CIP}. Scale bar: 50 μm. **(E)** The graph represents the quantification for the percentage of HUVEC cells staining positive for BrdU. The data represent the mean ± SEM of 3 independent experiments. Two-tailed Student´s t-test was used as test. **(F)** F9 quantitative ELISA measured in the conditioned media of HUVEC treated or not with Palbo for 7 days. Data represent the mean ± SEM of 4 independent experiments. Two-tailed Student´s t-test was used. **(G)** *F9* mRNA levels analysed by qPCR in in SKMEL28 treated with 500nM Palbo for 20 days. Please note data presented here are the same as SKMEL28 in **Figure 5D**. Student t-test statistical analysis performed. **(H)** SKMEL28 melanoma cells were treated with 10μg/mL of recombinant F9 (rF9) twice for 6 days. Ki67 and p21^{CIP} levels were determined by IF. Data show the mean ± SEM of 3 independent experiments. Two-tailed Student´s t-test was used as statistical analysis. Related to **Figure 3**.

Figure S4

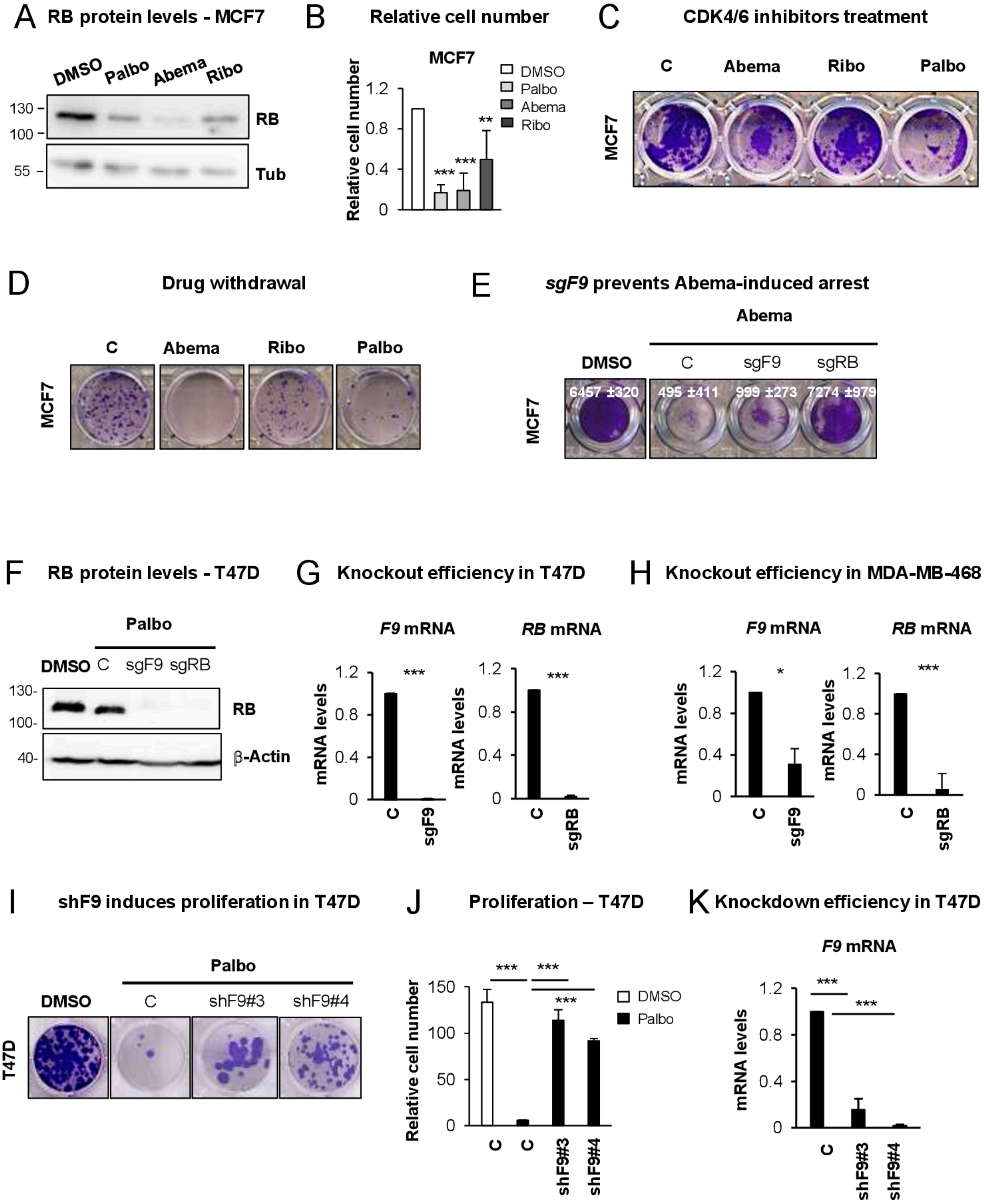

A RB protein levels - MCF7
DMSO
Palbo
Abema
Ribo
130
100
55
RB
Tub
B Relative cell number
MCF7
Relative cell number
1.2
0.8
0.4
0
DMSO
Palbo
Abema
Ribo
***
***
**
C CDK4/6 inhibitors treatment
C
Abema
Ribo
Palbo
MCF7
D Drug withdrawal
C
Abema
Ribo
Palbo
MCF7
E sgF9 prevents Abema-induced arrest
Abema
DMSO
C
sgF9
sgRB
6457 ±320
495 ±411
999 ±273
7274 ±979
MCF7
F RB protein levels - T47D
Palbo
DMSO
C
sgF9
sgRB
130-
100-
40-
RB
β-Actin
G Knockout efficiency in T47D
F9 mRNA
RB mRNA
mRNA levels
1.2
0.8
0.4
0
***
C
sgF9
sgRB
H Knockout efficiency in MDA-MB-468
F9 mRNA
RB mRNA
mRNA levels
*
***
C
sgF9
sgRB
I shF9 induces proliferation in T47D
Palbo
DMSO
C
shF9#3
shF9#4
T47D
J Proliferation – T47D
Relative cell number
150
100
50
0
DMSO
Palbo
***
C
shF9#3
shF9#4
K Knockdown efficiency in T47D
F9 mRNA
mRNA levels
1.2
0.8
0.4
0
***
C
shF9#3
shF9#4

Figure S4. CDK4/6 inhibitors response in a variety of cancer cell lines. (A) Immunoblot for RB in MCF7 cells treated with Palbo, Abema and Ribo show a decrease in the levels of RB confirming a proliferative arrest. **(B)** Relative cell number quantified after MCF7 cells were treated with 1μM of different CDK4/6 inhibitors (Palbociclib, Abemaciclib, Ribociclib). Data show the mean ± SD of 5 independent experiments. Two-tailed student's t-test analysis was performed. **(C)** Clonogenicity assay shows the proliferation rate of MCF7 cells after 14 days treatment with 1μM Abema, 1μM Palbo and 1μM Ribo. Representative experiment of 2 biological replicates. **(D)** Crystal violet staining showing the effect on proliferation after 6 days treatment with Palbo, Abema and Ribo. After drug withdrawal, cells were washed and the experiment was stopped 20 days after the initial treatment. Representative experiment is shown. **(E)** Crystal violet staining for colony formation assay in MCF7 cells expressing sgF9 or sgRB treated with 1μM Abema for 20 days. Representative experiment of 3 independent experiments is shown. Quantifications shows mean ± SD for relative cell number. **(F)** Representative western blot showing RB knockout in T47D cells. β-actin was used as a loading control. Blot representative of 3 independent experiments. **(G)** *F9* and *RB* mRNA levels were determined by qPCR in T47D cells expressing sgF9 or sgRB. Data show the mean ± SEM of 3 independent experiments. Two-tailed student' t-test analysis was performed. **(H)** Knockout efficiency for sgF9 and sgRB in MDA-MB-468 cells. mRNA levels were determined by qPCR. Data show the mean ± SEM of 3 biological replicates. Two-tailed student's t-test analysis was performed. **(I)** Crystal violet staining showing the proliferative rate of T47D cells expressing two independent shRNA targeting F9 (shF9#3 and shF9#4) treated with 1μM Palbo for 20 days. Representative experiment of 3 biological replicates. **(J)** Proliferation rate of T47D expressing shF9#3 and shF9#4 treated with 1μM Palbo for 20 days. The data represent the mean ± SEM of 7 independent experiments. One Way ANOVA with Dunnett's multiple comparisons to Palbo C sample was performed. **(K)** qPCR analysis for the levels of *F9* mRNA in T47D cells expressing shF9#3 and shF9#4. Data show the mean ± SEM of 3

independent experiments. Two-tailed student' t-test analysis was performed. See also to **Figure 4**.

Figure S5

A **Panel of 22 cell lines tested**

Renal	**ACHN**
Melanoma	**SKMEL28**
Breast	**MCF7**
Brain	**U87MG**
Liver	**SNU-387**
Colon	**HT-29**
Melanoma	**SKMEL2**
Renal	**A498**
Pancreatic	**Capan-2**
Prostate	**PC3**
Lung	**A549**
Colon	**HT-116**
Melanoma	**SKMEL5**
Bladder	**HT-1376**
Breast	**BT-549**
Bladder	**HT-1197**
Ovarian	**SKOV-3**
Ovarian	**OVCAR-3**
Brain	**U118MG**
Lung	**NHI-23**
Pancreatic	**PANC1**
Prostate	**DU-145**

Responding to ≥ 2 CDK4/6 inhibitors

B **Cell number in cells treated with CDK4/6i**

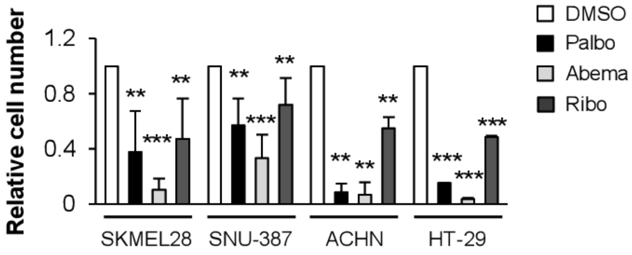

C **Continuous drug treatment**

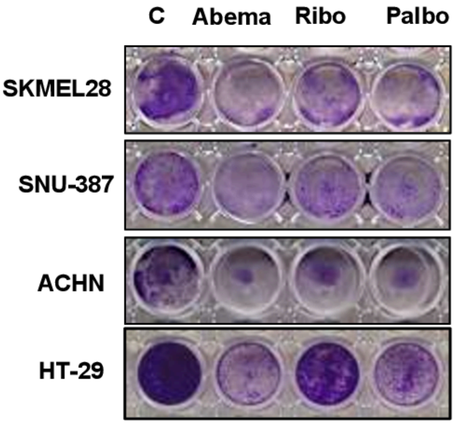

Figure S5. Response of other cancer cell lines to different CDK4/6 inhibitors

(A) Panel of cancer cell lines used in the Primary Screen to determine the efficacy of increasing concentrations of other CDK4/6 inhibitors on proliferation. The cell lines highlighted in orange are the ones that responded to two or more inhibitors. **(B)** Quantification of relative cell number from 8 different cancer cell lines selected for the Secondary Screen that responded to more than two CKD4/6 inhibitors. 1μM CDK4/6 inhibitor concentration was used. Data show the mean ± SD of 5 independent experiments for SKMEL28 and SNU-387 and 2 for HT-29 and ACHN. Student's t-test analysis was performed. **(C)** Crystal violet staining showing the effect on proliferation of Abema, Ribo and Palbo in the selected cancer cell lines after 14 days of continuous drug treatment. Related to **Figure 5**.

Figure S6

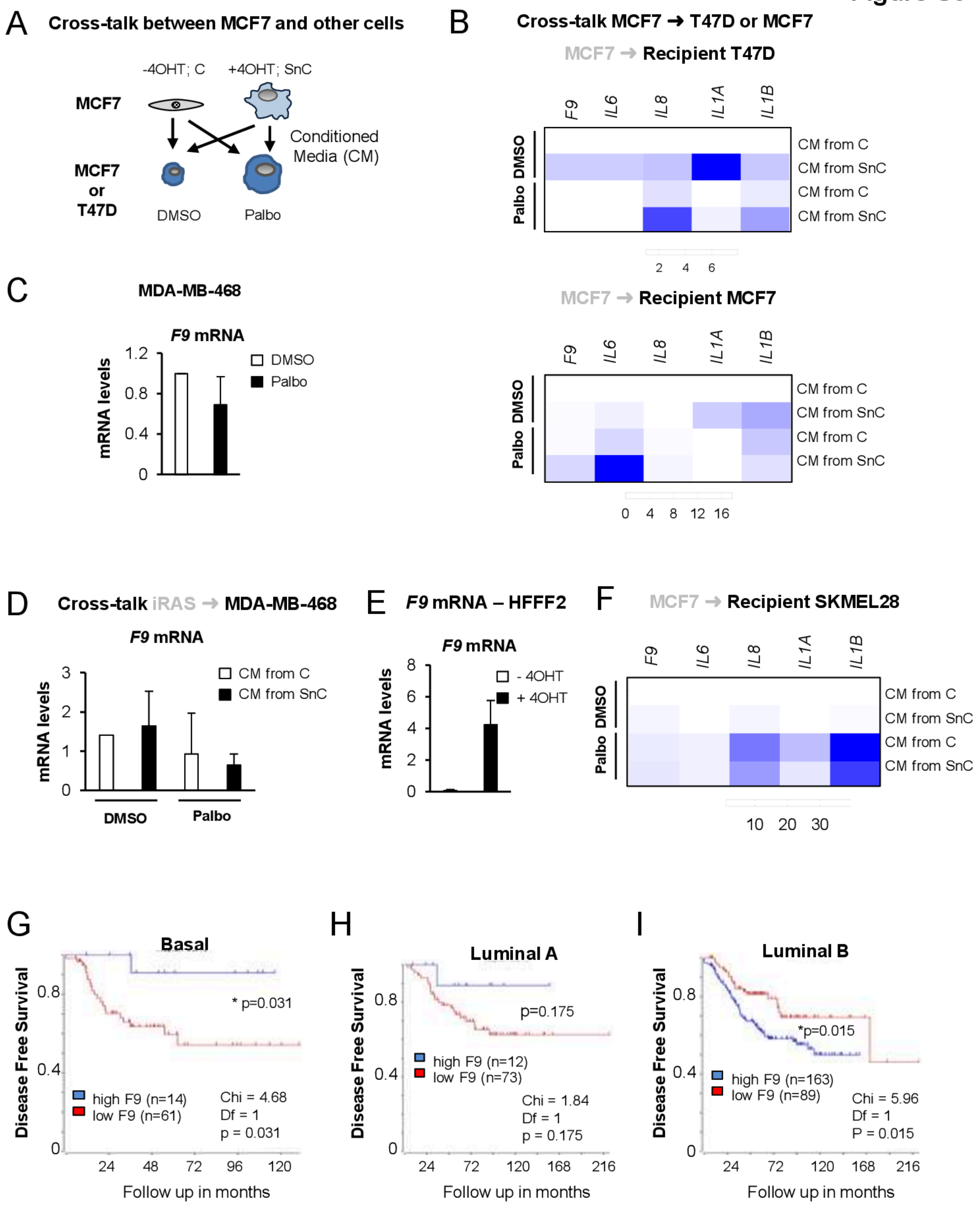
A
Cross-talk between MCF7 and other cells
-4OHT; C
+4OHT; SnC
MCF7
Conditioned Media (CM)
MCF7 or T47D
DMSO
Palbo
B
Cross-talk MCF7 ➜ T47D or MCF7
MCF7 ➜ Recipient T47D
F9
IL6
IL8
IL1A
IL1B
DMSO
Palbo
CM from C
CM from SnC
CM from C
CM from SnC
2 4 6
MCF7 ➜ Recipient MCF7
0 4 8 12 16
C
MDA-MB-468
F9 mRNA
mRNA levels
1.2
0.8
0.4
0
DMSO
Palbo
D
Cross-talk iRAS ➜ MDA-MB-468
F9 mRNA
mRNA levels
3
2
1
0
CM from C
CM from SnC
DMSO
Palbo
E
F9 mRNA – HFFF2
F9 mRNA
mRNA levels
8
6
4
2
0
- 4OHT
+ 4OHT
F
MCF7 ➜ Recipient SKMEL28
10 20 30
G
Basal
Disease Free Survival
0.8
0.4
0
* p=0.031
high F9 (n=14)
low F9 (n=61)
Chi = 4.68
Df = 1
p = 0.031
24 48 72 96 120
Follow up in months
H
Luminal A
0.8
0.4
0
p=0.175
high F9 (n=12)
low F9 (n=73)
Chi = 1.84
Df = 1
p = 0.175
24 72 120 168 216
Follow up in months
I
Luminal B
0.8
0.4
0
*p=0.015
high F9 (n=163)
low F9 (n=89)
Chi = 5.96
Df = 1
P = 0.015
24 72 120 168 216
Follow up in months

Figure S6. Influence of the intercellular cross talk between different cell cultures. (A) Schematic representation of the experimental design to identify the cross-talk between Palbo-induced MCF7 cells and other cancer cell lines. (**B**) Quantification of different SASP and *F9* mRNA levels in recipient cells (MCF7 and T47D) treated with the CM from MCF7 cells treated with Pablo or not. (**C**) mRNA levels of *F9* in MDA-MB-468 breast cancer cell line. Data represent the mean ± SEM of 3 independent experiments. (**D**) Cross-talk between iRAS human primary fibroblasts undergoing senescence or not and treatment of MDA-MB-468 with their CM. Data represent the mean ± SEM of 3 independent experiments. (**E**) *F9* mRNA levels for HFFF2 iRAS fibroblasts undergoing senescence upon +4OHT treatment. Data show the mean ± SEM of 3 independent experiments. (**F**) *F9* and other SASP mRNA levels in SKMEL28 human melanoma cancer cell lines exposed to the conditioned media of proliferative or senescence MCF7 cells. (**G, H, I**) Kaplan-Meier survival curves for different breast cancer subtypes: (**G**) Basal, (**H**) Luminal A and (**I**) Luminal B showing the expression levels of F9 high (blue) or low (red) and disease-free survival probability[30]. Dataset calculated with R2.
